# Ebstein Anomaly Successfully Treated With Levosimendan Postoperatively in a 60-Year-Old Female: A Case Report

**DOI:** 10.7759/cureus.66340

**Published:** 2024-08-06

**Authors:** Noora Aljalahma, Heba Alkoheji, Helen Saunders, Habib Tareif

**Affiliations:** 1 Surgery, Military Hospital, Royal Medical Services, Riffa, BHR; 2 Internal Medicine, Military Hospital, Royal Medical Services, Riffa, BHR; 3 Anesthesiology, Mohammed Bin Khalifa Bin Salman AlKhalifa Cardiac Center, Awali, BHR; 4 Cardiothoracic Surgery, Mohammed Bin Khalifa Bin Salman AlKhalifa Cardiac Center, Awali, BHR

**Keywords:** echo, atrial septal defects, cone procedure, asd repair, levosimendan, elderly, ebstein’s anomaly

## Abstract

Ebstein anomaly is a congenital heart disease that is considered rare and mostly found in pediatrics population. Symptoms in adults vary depending on the degree of the valve displacement and include difficulty breathing, palpitations, stroke, or even fatigue. However, if it occurs in the elderly, they end up with a good prognosis. A novel calcium sensitizer “levosimendan” has been used perioperatively in heart valve replacement to improve the long-term prognosis of patients. The use of the drug has been shown to reduce postoperative mortality in patients with reduction in ejection fraction. We present the case of a 62-year-old female, a known case of hypothyroidism, bronchial asthma, gastroesophageal reflux disease, and recent diagnosis of Ebstein anomaly, who underwent tricuspid valve repair and atrial septal defect repair on being symptomatic, in addition to the successful use of a novel positive inotropic drug with decrease in the intensive care unit stay.

## Introduction

Ebstein anomaly is a rare congenital heart disease involving malformation of the tricuspid valve (TV) and the right ventricle (RV). It is characterized by displacement of the valve leaflets being partly attached to the annulus and partly to the RV endocardium, causing TV regurgitation and right heart enlargement. The condition involves a wide variety of presentation, with those presenting at infancies with no survival and those presenting as adults incidentally with good prognosis. The American Heart Association [1] noted that the condition usually presents with other anomalies including atrial septal defects (ASDs), like in our patient. Some patients might present with ventricular septal defect, pulmonary stenosis, and patent foramen ovale. Furthermore, arrhythmias and accessory pathways are relatively common in these patients [2].

The prevalence of Ebstein anomaly has been reported to be around 0.005% of live births. The incidence in the general population is approximately 1.20 to 5 per 100,000 live births. A European registry-based study found that the total prevalence of Ebstein anomaly increased over time from 0.29 to 0.48 per 10,000 births between 1982 and 2011. This could be due to better and earlier diagnosis. The study found that Ebstein anomaly may be associated more frequently with maternal health problems rather than, as widely believed, the use of lithium or benzodiazepines. This might be important to consider especially in this era where mental health problems are on a rise [1-3].

Surgical management is indicated in Ebstein anomaly. However, one of the possible complications occurring following cone procedure for Ebstein anomaly is right ventricular failure. This is predicted via right ventricular dilation with a measurement of >200 mL/m2, reduced right ventricular ejections fraction <40%, and age more than 50 years. The presence of two or more risk factors is associated with early mortality and morbidity [4].

The aim of this case report is to share the unique presentation of Ebstein anomaly with concurrent ASD in an elderly patient. This report also serves to inform and educate the medical community with success from both surgical and anesthetic points of view with the introduction of a novel calcium sensitizer such as levosimendan.

## Case presentation

A 62-year-old female, a known case of hypothyroidism, bronchial asthma, gastroesophageal reflux disease, past surgical history of right mastectomy 10 years ago, and a recent diagnosis of Ebstein anomaly, presented to Mohamed Bin Khalifa Bin Salman Al Khalifa Specialist Cardiac Center (MKCC).

The patient was initially asymptomatic and had been on her regular annual follow-up when suddenly she developed difficulty in breathing. Her blood pressure was 127/80 mmHg, heart rate was 82 beats/minute, and oxygen saturation was 93% on room air. She underwent several investigations. In 2022, the patient underwent transthoracic echocardiography (TTE), which showed an enlarged right atrium (RA), arterialized RV, and apically displaced TV with severe tricuspid regurgitation (TR). Also, the Holter monitor test showed an average heart rate of 93 bpm with no significant arrhythmias. A six-minute walk test (6MWT) showed that 323.7 meters were covered out of 498.2 meters, after which the patient developed shortness of breath, and oxygen saturation dropped to 80%.

**Figure 1:**
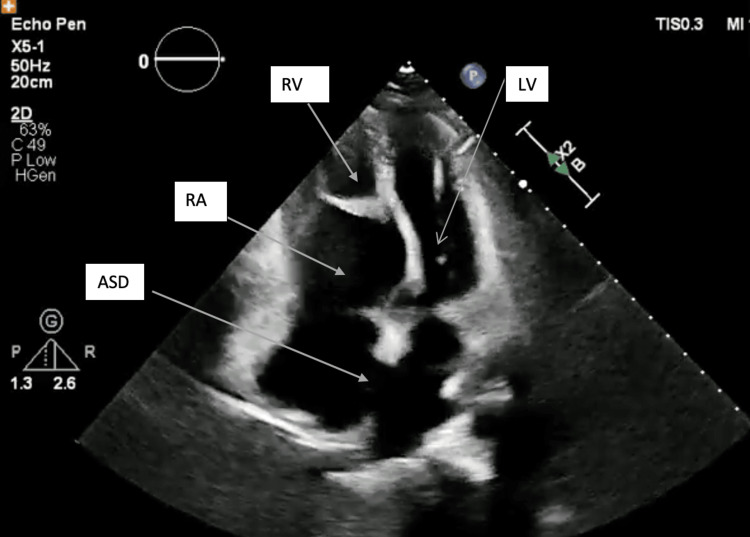
Apical displacement of the tricuspid valve, atrialization of the inlet of the RV, and secundum ASD ASD, atrial septal defect; LV, left ventricle; RA, right atrium; RV, right ventricle

In January 2023, after the onset of symptoms, she underwent transesophageal echocardiogram (Figure 1), which showed Carpentier (grade B/C) Ebstein anomaly with severe tethering, dysplasia, and apical displacement of septal (2.4 cm/m^2^) and posterior tricuspid leaflets, moderate-to-severe TR with pulmonary arterial systolic pressure (PASP) 35 mmHg, significant arterialization of distal and thin RV chamber, and relatively small functional RV with normal thickness. RV fractional area change was 35%. A large ostium secundum ASD with left-to-right shunt was also noted. Left ventricle (LV) size was small, with subaortic bulge and paradoxical basal left ventricular summit motion toward LV, which resulted in dynamic left ventricular outflow tract turbulence without any significant intraventricular gradient. Despite these abnormalities, the LV systolic function was visually preserved. Additionally, the echocardiogram revealed bileaflet mitral valve prolapse with mild late systolic mitral regurgitation. After being diagnosed with severe TR, the patient was scheduled for cone procedure and ASD closure.

Preoperatively, the patient was assessed by an anesthesiologist, cardiologist, and physiotherapist. She received antibiotic prophylaxis perioperatively. The patient then underwent TV repair and ASD on September 4, 2023. The approach was made under general anesthesia via median sternotomy and vertical pericardiotomy. Purse string suture was applied to the ascending aorta and the RA. Heparin was given, followed by cannulation of the ascending aorta and both cavae. Cardiopulmonary bypass was established with core cooling to 28 degrees Celsius. The ascending aorta was cross-clamped, and cardioplegia was delivered antegradely. TV repair was performed subsequently. For instance, the RA was opened, and the valve was inspected. Cone repair was performed, encompassing five aspects. First, the leaflets were delaminated, meaning they were circumferentially detached, leaving a hinge point of attachment for the septal leaflet. Additionally, plication of the atrialized portion of the right ventricle was achieved using pledgeted 4-0 Prolene sutures, and annular plication was performed to further stabilize the repair. Moreover, cone reconstruction was performed by re-attaching the tricuspid leaflet to the new annulus using 6-0 Prolene sutures; the septal leaflet was also augmented with a pericardial patch. Finally, the commissure between the anterior and posterior leaflets was sutured with 6-0 Prolene. the ASD was closed using autologous pericardial patch and 6-0 Prolene stitch. The RA was closed with 5-0 Prolene, while the left atrium, LV, and aorta were de-aired. After the cross-clamp removal, the heart resumed it’s activity from ventricular fibrillation to normal sinus rhythm.

**Figure 2:**
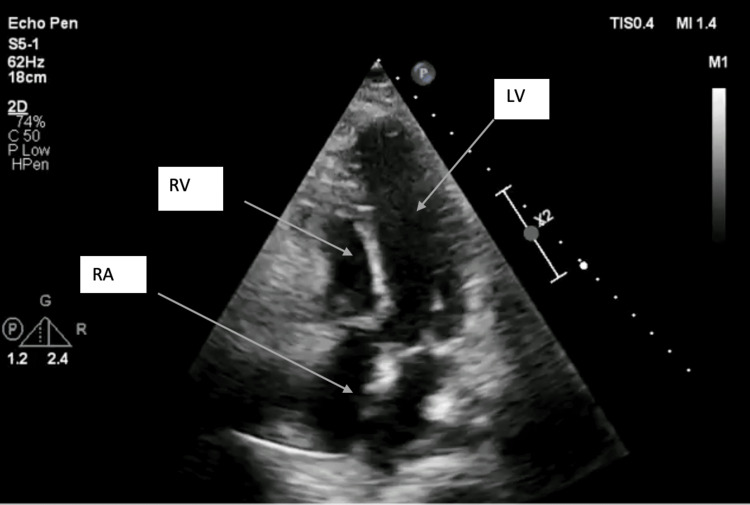
Post-procedure TTE showing successful cone repair LV, left ventricle; RA, right atrium; RV, right ventricle; TTE, transthoracic echocardiogram

Post-procedure TTE was performed to check the repair (Figure 2). Bypass time was 120 minutes, cardiopulmonary bypass was discontinued, and heparin was replaced with protamine. There was no hemodynamic instability while discontinuing bypass. Levosimendan 1.2 mL was started with a dose of 0.1 mcg/kg/min. The pericardium was then approximated using polytetrafluoroethylene membrane, and the sternum was closed using 8 Ethibond sutures. Subcutaneous tissue was closed in two layers using polydioxanone suture and Vicryl. Subcuticular Vicryl was used for skin closure.

Postoperatively, the patient was shifted to the cardiac intensive care unit and was hemodynamically stable. She was started on noradrenaline 0.01 mic/kg/mL for better prognosis. Her vitals were stable, with a blood pressure 140/80 mmHg, heart rate of 87 beats per minute, SpO_2_ of 98% on mechanical ventilation. She was on pleural and pericardial drain with left ventricular pacing wire. She was also started on furosemide, aspirin 75 mg, and proton pump inhibitor prophylaxis. The day after the procedure, the patient was shifted to the ward. She was placed on high-flow nasal cannula therapy, receiving 40% oxygen at a flow rate of 40 liters per minute, without the need for inotropic support. On day 3 after surgery, the patient was placed on 4 liters of nasal cannula. On day 4 of surgery, the nasal cannula was weaned to 2 liters, and the chest drain and pacing box were removed. Her postoperative TEE showed a left ventricular ejection fraction (LVEF) of 70-75%. Mean gradient across the TV 5 was mmHg, there was mild TR, interatrial septum appeared normal and intact, and there was no evidence of interatrial shunting or pericardial collection. She was doing well and hence discharged in a stable condition on September 9, 2023. The patient was restarted on Concor and spironolactone. She was discharged on the fifth day, maintaining oxygen saturation on room air. Her discharge medications included furosemide, pantoprazole, ipratropium, spironolactone, linagliptin, and clopidogrel.

Furthermore, she was followed up regularly in the cardiac center; she was asymptomatic, doing well, and adherent to her prescribed medications.

## Discussion

The clinical presentation of Ebstein anomaly depends on the extent of TV leaflet distortion, the degree of the right ventricular dilation and its dysfunction, and the presence or absence of right-to-left shunt. Ebstein anomalies have a wide spectrum of presentation. For instance, in neonates, the anomaly may be very severe, causing the neonate to not survive infancy due to severe cardiomegaly, heart failure, or pulmonary hypoplasia. Milder distortion of the anatomy with mild-to-moderate TR may be diagnosed incidentally in adulthood or may present with arrhythmia or paradoxical embolic event. On the other hand, those who have marked displacement of the valve causing severe regurgitation, like our patient, may present with right-sided heart failure, elevated right atrial pressure, and significant stenosis due to the presence of the shunt. Symptoms may include exertional dyspnea and fatigue, while cyanosis, stroke, transient ischemic attack usually occur in the presence of a shunt [1].

Atrial tachyarrhythmias are more frequent in adolescents and adults, presenting in approximately 20-30% of the cases. Physical examination is a trivial part of any cardiac condition. On auscultation of a patient with suspected Ebstein anomaly, systolic murmur is a common finding indicative of TV regurgitation. The murmur increases with inspiration and may be concurrent to a mid-diastolic murmur. Of note, the murmur is not an explicit finding in adults. In the setting of TV regurgitation, we expect to have a distended jugular vein with a prominent V wave. However, the V wave is often absent in those patients due to the dilatation of the RA with the atrilization of the ventricle. If the patient had a right bundle branch block, the first and second heart sounds are widely split and an early systolic click may be present [1].

A retrospective study conducted in MKCC in the Kingdom of Bahrain from the period 2000 to 2016 diagnosed nine cases of Ebstein anomaly with a rate of 0.4% of the community and 0.03 per 1,000 live births. Moreover, another retrospective study conducted by Luu et al. found that 51 patients were diagnosed with Ebstein anomaly between 2000 and 2013. Interestingly, the mean age of diagnosis was 21 years. The commonest anomaly seen in association with Ebstein anomaly is ASD [5,6].

In elderly individuals, the diagnosis of Ebstein anomaly is typically made through a combination of clinical evaluation and imaging studies. The initial imaging of suspicion of Ebstein anomaly will be TTE. This is the key test of diagnosis, with a key diagnostic finding being apical displacement of the septal tricuspid surface area (by ≥8 mm/m^2^) compared to the anterior mitral valve leaflet. The degree of displacement can also establish the severity of the presentation. It can quantify the severity of TR and the changes in right heart volume. TTE is also useful to exclude other associated anomalies. Cardiac magnetic resonance (CMR) imaging can also be particularly useful in quantifying the size of the RA and the RV and assessing the anatomy, which can all be challenging with echocardiography alone. CMR and echocardiogram are helpful pre- and postoperatively. Additional testing may be performed, including exercise testing with oximetry or cardiopulmonary exercise. This can help identify the exercise capacity and any potential desaturation. Moreover, pulse oximetry can be used for risk stratification. Additionally, electrocardiogram (ECG) and annual 24-hour ambulatory ECG can be ordered to assess arrhythmias. Sometimes, electrophysiological study may be required as part of further evaluation. Finally, if the patient is suspected to have pulmonary hypertension, cardiac catheterization is suggested as per UpToDate guidelines [2].

The treatment for Ebstein anomaly in adults is multifaceted and depends on the severity of the condition and the presence of symptoms. Surgical management is the mainstay of treatment in Ebstein anomaly patients. According to the 2018 American College of Cardiology/American Heart Association adult congenital heart disease guidelines, patients with heart failure symptoms, deterioration of the exercise capacity, and progression of the right ventricular dysfunction on imaging are candidates for surgery. Patients with cyanosis, paradoxical embolism, atrial tachyarrhythmia, and progressive RV dilation on imaging may benefit from surgery. Finally, any patient with severe TR who is fit for surgery and has a low operative risk of <1 % are advised for surgical repair [1,7].

Medical management is usually used for temporary stabilization of the patient prior to surgery if the patient is symptomatic. Antithrombotic therapy for patients with a known shunt without other standard indications is controversial. Its role has not been established; thus, risk stratification is required. Furthermore, endocarditis prophylaxis is recommended for cyanotic patients or patients with prosthetic cardiac valves [7].

The options for surgical management in the literature included TV repair with different techniques such as Danielson or Carpentier technique. Cone-type reconstruction is another technique in which there will be mobilization of all the tricuspid leaflets and re-anchoring it to the true level of the annulus with plication of the inferior wall of the RV. TV replacement can be performed if a repair is not possible. In these instances, a biologic prosthesis with porcine is preferable to prevent the high incidence of thrombosis with mechanical prosthesis or with pericardial valve prosthesis. In some cases, additional interventions may be required. For instance, a bidirectional cavopulmonary shunt (Glenn procedure) can be used if the patient is hemodynamically unstable or has severe right ventricular dilation or severe dysfunction with preserved left ventricular function and pressure [1,7,8].

While exploring the literature, we found that cone reconstruction had been associated with improvement of the biventricular function and positive signs of biventricular remodeling. The operation results in improvement of the TR and right ventricular size and increases right ventricular fractional area change and left ventricular volume. However, a possible temporary complication is the initial reduction in right ventricular ejection fraction postoperatively, but this effect rebounds by six months of the operation [9,10].

A study conducted by Burri et al. [11] found the cone procedure has been associated with better success of the repair and lower rates of recurrent TR. The American Heart Association [12] suggests that the choice of repair method relies on numerous factors, including the surgeon's experience and choice, the mechanism of the regurgitation, and the morphology of the valve.

We found a few case reports in the literature of elderly patients diagnosed with Ebstein anomaly. For instance, an 86-year-old male smoker, known case of hypertension, was diagnosed recently with coronary artery disease associated with Ebstein anomaly and presented with two months of typical chest pain on exertion and New York Heart Association (NYHA) class II. The patient underwent TTE, which showed mild-to-moderate TR and dilatation of both the right atria and ventricle, features of Ebstein’s associated a patent foramen ovale. Ejection fraction was noted to be 45-50%. Upon the patient’s wishes, surgery was not performed, and anti-ischemic medications (beta blocker, aspirin, angiotensin-converting enzyme inhibitor, and statin) with anti-coagulations were started. On his follow-up appointments, his chest pain was relieved and he could tolerate exercise. The patient characteristics were different from our patient, with ejection fraction being reduced. Even though surgery was recommended in most patients, this patient had NYHA class II, shared decision with the patient is the mainstay after informed consent [13].

Additionally, Kawase et al. reported a case of an elderly female patient with Ebstein anomaly who was asymptomatic and stated that the operation decision was made because the patient had features of severe TR and right ventricular dilatation, which resulted in success of the operation [14].

It is widely known that positive inotropic drugs are the most beneficial drugs for improvement of cardiac function postoperatively in patients undergoing cardiac surgery. However, these drugs have been shown to cause adverse effects leading to higher risk of myocardial ischemia and arrhythmias. Fortunately, a new drug has been introduced that has been shown to not increase myocardial oxygen consumption and thus avoid the adverse effects seen in other positive inotropic agents. Clinical trials have shown that levosimendan had its greatest effects in patients having diminished LVEF [15].

The effects of levosimendan on right ventricular function have been studied in a systematic review and meta-analysis. Evidence have shown that after 24 hours of administration of levosimendan, the right ventricular fractional, tricuspid annular peak systolic velocity, tricuspid annular plane systolic excursion, and cardiac output all increased with decreased in pulmonary artery pressure, indicating favorable outcomes to those with pre-existing right heart dysfunction [16].

To our knowledge, no case reports have been found in the literature showing the effects of levosimendan in a patient with right ventricular failure undergoing cardiac surgery.

## Conclusions

Ebstein anomaly is a condition that is mostly known to occur in the younger population, but it can also appear in the elderly, as in our patient. The diagnosis of Ebstein anomaly can be made through a combination of clinical suspicion and the initial investigation with TTE. Surgical management is the principal treatment of this condition, showing promising results, as in our patient. Cardiac surgery can be challenging, and levosimendan (a novel calcium sensitizer) has been used peri-operatively to improve the prognosis in patients with reduced LVEF. However, our patient had right ventricular dysfunction, which we managed with levosimendan, which resulted in a positive impact on the long-term prognosis and the complete recovery of the patient. Overall, this case report draws attention to this condition mainly in this region as there is lack of reporting cases in the Middle East and also informs the medical community on how to treat this condition if anyone encounters a similar case.
